# Acquired Haemophilia A in the Elderly: Case Reports

**DOI:** 10.1155/2010/927503

**Published:** 2010-03-03

**Authors:** Antonella Tufano, Antonio Coppola, Anna Guida, Ernesto Cimino, Angela Maria De Gregorio, Anna Maria Cerbone, Giovanni Di Minno

**Affiliations:** Regional Reference Centre for Coagulation Disorders, Department of Clinical and Experimental Medicine, “Federico II” University Hospital, 80131 Naples, Italy

## Abstract

Acquired hemophilia A (AHA) is a very rare disease, caused by the development of autoantibodies, directed against circulating factor VIII of coagulation. Age distribution is bimodal, with a first peak occurring among young women in the postpartum period, and a second major peak of incidence among elderly patients in whom it is frequently associated with malignancy and drugs. This disease often represents a life-threatening bleeding condition, especially in the elderly, thus requiring a prompt therapeutic intervention, including control of acute bleeding and eradication of the inhibitor by immunosuppressive therapy. The diagnosis of AHA should be considered in any elderly patient who presents with bleeding and prolonged activated Partial Thromboplastin Time. Moreover, the coexistence of a series of underlying diseases associated with AHA should be always searched for. An early recognition and an adequate treatment of this coagulation disorder and of the possible associated diseases play a significant role for a favourable outcome, but concomitant morbidities in the elderly may limit aggressive therapy and may complicate the clinical scenario. We report 3 consecutive elderly patients successfully treated with recombinant activated factor VII and standard immunosuppressive regimens, with remission of the disease.

## 1. Introduction

Acquired haemophilia A (AHA) is a very rare disease, with an estimated annual incidence between 1.3 and 1.5 per million per year [1–6]. This potentially life-threatening bleeding disorder is caused by the development of autoantibodies, generally IgGk4, directed against circulating factor VIII (FVIII) of coagulation. Age distribution is bimodal, with a first peak occurring among young adults, due to cases in women in the postpartum period, and a second major peak in elderly patients in whom it is frequently associated with malignancy and drugs, and very difficult to manage due to the comorbidities and the “fragility” of the older subjects [7]. Other associated conditions are: autoimmune disorders, hematologic malignancies, inflammatory bowel diseases, dermatologic disorders, respiratory diseases, diabetes mellitus, and hepatitis B and C infections. The treatment of the underlying diseases often eradicates the coagulation autoantibodies [1–6, 8]. In most patients, however, FVIII autoantibodies are idiopathic [1–6].

The pattern of bleeding in AHA differs from that in congenital haemophilia A. Bleeding tends to occur in soft tissue, muscle, retroperitoneal space, and gastrointestinal or genitourinary tracts. In contrast with patients with congenital haemophilia A, patients with haemarthroses are rare. Iatrogenic bleeding is also common. Rates of mortality from acquired haemophilia up to 44% have been reported, with most deaths occurring in the first few weeks [6]. Thus a prompt recognition of this disorder and an early and aggressive treatment are mandatory, as diagnostic delays or inadequate treatments are associated with high mortality rates.

Diagnosis may be challenging, since the patient will have no personal or family history of bleeding disorders. However, in any patient who presents with recently-onset severe or deep tissue bleeding and an unexplained isolated prolonged activated Partial Thromboplastin Time (aPTT), AHA should be considered [1–6] (Table 1). The presence of circulating inhibitors is confirmed by the mixing test, showing the lack of correction of the aPTT when a mixture of equal volumes of patient and normal plasma is incubated at 37° for 2 hours or longer. An isolated low FVIII:C level is suggestive of AHA [1–6, 9]. The diagnosis is then confirmed by a Bethesda positive assay for FVIII inhibitor titre: 1 Bethesda Unit (BU)/mL is the quantity of inhibitor that neutralizes 50% of clotting factor activity in normal plasma) [1–6, 9]. In most cases, however, the bleeding phenotype does not correlate with laboratory assessment (FVIII level and inhibitor titre) [1–6].

Treatment of AHA focuses on 2 goals: control of acute bleeding and immunosuppressive therapy to eradicate FVIII inhibitors. Acute bleeding episodes are usually treated with bypassing agents and good efficacy is seen with both recombinant activated (rFVIIa) and activated prothrombin complex concentrates (aPCCs). Eradication of the autoimmune inhibitor antibody with immunosuppression is indicated as soon as the diagnosis has been confirmed, because patients remain at risk of potentially fatal bleeding until the inhibitor is suppressed. Steroids, alone or in combination with cyclophosphamide or azathioprine, induce remission in about 70% of the patients. [1–6]. In nonresponders, alternative approaches have been proposed, in particular, using rituximab [4, 10–14]. This chimeric human/mouse monoclonal antibody directed at the CD20 antigen expressed in mature-B and pre-B lymphocytes induces B-cell depletion rapidly and completely. However, disease activity has been seen to recur at the time of B-cell repopulation, so reteatment is usually necessary. Outcomes and adverse effects of long-term B-cell depletion are unknown, so caution should be eserted, especially in patients with a good prognosis [14, 15].

We report clinical features and treatment of three patients with a history of bleeding occurring in the elderly in whom AHA was diagnosed.

## 2. Case Reports

**Case 1**

A 70-year-old man presented with large posttraumatic left leg haematoma. The patient had a history of coronary heart disease (acute myocardial infarction treated with percutaneous transluminal coronary angioplasty and stenting, 10 years earlier), therefore he was on chronic antiplatelet treatment (aspirin 100 mg/day). About 3 years earlier, because of haematuria, he performed an abdominal ultrasonography (US) that revealed a renal mass confirmed at computed tomography (CT) scan, but the patient did not perform further controls. As gastrointestinal bleeding (melena, due to multiple peptic ulcers) occurred one year earlier, treated with endoscopic haemostasis and blood transfusion, aspirin was stopped and substituted for clopidogrel. One month later an US confirmed the renal neoplasm (diameters approximately 12 cm and irregular burden, with a central necrotic area); for this reason he was administered sunitinib, scheduled for a nine-cycle therapy. Surgical treatment of the neoplasm was not considered in this patient because of his high bleeding tendency. After a few weeks, because of a rectal bleeding, he was admitted in an emergency care unit: clopidogrel was stopped and laboratory tests revealed prolonged aPTT, normal platelet counts and anaemia. In the suspect of AHA, he was treated with rFVII (90 *μ*g/kg i.v. bolus) with complete resolution of bleeding. Further investigations of the coagulation abnormality were not performed at that time.

Because of the leg haematoma and lower back pain, he was finally referred to our hospital. On admission, coagulation tests confirmed the prolonged aPTT (92 seconds, normal 26–44), not corrected at mixing test, with normal prothrombin time (PT), international normalized ratio (INR), and bleeding time (Ivy, 3 minutes, normal values <7). Haemoglobin level was 9.4 g/L and platelet count was 260 × 10^9^/L. Abdominal US showed the presence of right iliopsoas and gluteal haematomas. A CT scan confirmed the presence of the renal neoplasm and of the muscle bleeds. FVIII:C was 9.3% and the FVIII:C inhibitor titre was 3.5 BU/mL. AHA was, therefore, diagnosed. He was treated with a single rFVII 90 *μ*g/kg i.v. bolus with a reduction of back pain and with an apparent cessation of bleeding (stable haemoglobin level and dimensions of haematomas at US follow-up). Immunosuppressive therapy with prednisone 1 mg/kg/day was started the day after admission. About two weeks later, aPTT was still prolonged, FVIII:C was 29%, and FVIII:C inhibitor titre was 2.1 BU/ml, thus cyclophosphamide 1 mg/Kg/day was added. Four weeks later, normalization of aPTT was obtained, inhibitor was 0.2 BU/ml, haemoglobin levels progressively improved and steroid therapy was continued for the following 4 weeks and then gradually discontinued. Cyclophosphamide was stopped 6 weeks after the eradication of the FVIII:C inhibitor. Since a sustained response was obtained, according to recent recommendations [5], aPTT, FVIII:C, and FVIII inhibitor were evaluated monthly, in the first six months, then every 3 months in the following six months and every six months thereafter. During a two-year follow-up the patient presented no further haemorrhage. Three months after the discharge, he started low dose aspirin (100 mg/day) for secondary prevention of his coronary heart disease. Sunitinib was continued. The neoplasm was assumed as the cause of AHA in this elderly patient.

**Case 2**

A 68-year-old man presented with multiple spontaneous haematomas (gluteal, neck, and lower limbs), in the absence of personal or family history of bleeding or clotting disorders. His past medical history included type 2 diabetes mellitus, arterial hypertension, prostatic hypertrophy, psoriasis, and arthrosis. At that time he was taking nonsteroidal anti-inflammatory drugs (NSAIDs) because of back pain. A previous haematoma of the left arm, after an effort, occurred approximately 6 weeks before the admission, when he also was on NSAID treatment; one month later he reported a gluteal and leg haematomas immediately after an intramuscular NSAID injection because of his recurrent back pain. A week later he presented with a wide neck haematoma unsuccessfully treated with tranexamic acid, therefore he was referred to our hospital. On admission, laboratory investigations revealed anaemia (haemoglobin 8.2 g/L) and normal platelet count (219 × 10^9^/L). Coagulation tests showed a moderately prolonged aPTT (54.8 seconds) with normal PT and INR. APTT was not modified after mixing test, lupus anticoagulant (LAC) was negative, FVIII:C was 16%, and FVIII inhibitor was 2.3 BU/ml, leading to diagnosing AHA. Factor IX, factor X, factor XI, factor XII, and von Willebrand Factor were normal, bleeding time was 7 minutes and 30 seconds.

The day after the admission, the patient had dyspnoea and haemoglobin levels further decreased (7.0 g/dl), thus transfusions with packed red blood cells were required (2 Units). A CT scan confirmed the presence of a large haematoma in the neck. Because of the reduction of haemoglobin levels and the harmful site of bleeding, a 90 *μ*g/kg rFVIIa i.v. bolus was administered and repeated after 3 hours with an apparent resolution of bleeding (stable haemoglobin values). Concomitantly, immunosuppressive therapy with prednisone 1 mg/kg/day was started. As spontaneous bruising of the upper limbs occurred and aPTT was still prolonged, cyclophosphamide 1.5 mg/kg/day was added. Clinical and laboratory features improved in the following two weeks, with a progressive normalization of the aPTT and the eradication of the FVIII inhibitor (undetectable inhibitor, normal FVIII:C). Since inhibitor eradication was obtained, cyclophosphamide was discontinued after 4 weeks and prednisone gradually stopped after 2 months. During a three-year follow-up, no further bleeding occurred. Psoriatic arthritis was diagnosed few months after the withdrawal of immunosuppressive treatment. This underlying immunologic disorder was therefore associated with AHA in this patient, in whom bleeding tendency had been triggered by NSAIDs.

**Case 3**

A 87-year-old woman presented with persistent rectal bleeding and large ecchymoses. Her past medical history included Hodgkin's disease about 10 years earlier, in apparent long-term remission, and type 2 diabetes mellitus.

On admission, laboratory investigations revealed severe anaemia (haemoglobin 6.7 g/L) and mild reduction of platelet count (125 × 10^9^/L). Coagulation tests showed a prolonged aPTT (99 seconds) not corrected at mixing test and normal PT and INR. LAC was negative, FVIII:C was 16%, and FVIII inhibitor was 33.2 BU/ml, confirming the diagnosis of AHA. Transfusions with packed red blood cells were required due to anaemia and the persistent haemorrhagic tendency, and a rFVIIa 90 *μ*g/kg i.v. bolus was administered, and repeated after three hours, with temporary cessation of bleeding. Prednisone was immediately started at doses of 1 mg/kg/day. One week later, she presented again decrease in haemoglobin level, with FVIII:C and FVIII inhibitor unchanged. The patient received packed red blood cells and another rFVIIa i.v. bolus. A CT scan revealed a large dorsal muscle haematoma, with no signs of recurrence of Hodgkin's disease. Further investigations led to exclude other malignant or autoimmune diseases. Therefore this case was assumed to be idiopathic AHA. During admission, she experienced other episodes of rectal bleeding with significant reduction of haemoglobin levels. In each occasion, she received single rFVIIa i.v. bolus, achieving haemostasis. After three weeks of prednisone treatment, aPTT was normalized and FVIII:C was 105%, with negative FVIII inhibitor. However, the patient presented septic fever and dyspnoea, with clinical signs of pneumonia and pleural effusion, and positive haemoculture for Staphylococcus Aureo. In spite of treatment with imipenem (500 mg thrice a day) and teicoplanine (200 mg once daily), the patient presented a rapid worsening of haemodynamic and respiratory condition, requiring orotracheal intubation and intensive care treatment. In this patient AHA was not fatal because of bleeding, but because of septic complications due to immunosuppressive therapy.

## 3. Discussion

AHA is a rare but life-threatening bleeding disorder, typically occurring in the elderly. According to recent data, the incidence in subjects aged <65 yrs is 0.28 per million per year, but increases up to 5.97 in those aged 65–85 yrs and to 16.6 in individuals older than 85 yrs [2]. On the whole, more than 80% of diagnosed patients are aged >60 yrs [2, 3]. Moreover, these data are likely to be underestimated because of undiagnosed and unreported cases. Therefore, the impact of AHA is not negligible in elderly patients, in whom the differential diagnosis with other age-correlated bleeding conditions should be also taken into account (Table 2) [16].

The diagnosis of AHA should be considered in any elderly patient who presents with bleeding and an isolated prolonged aPTT. The mixing test is a simple assay able to reveal the presence of inhibitors of coagulation [9]. Indeed, a prolonged aPTT may be attributable to coagulation factor deficiencies, lupus anticoagulant (LAC), or heparin therapy (Table 3). The presence of LAC may also be associated with a prolonged aPTT that is not corrected with normal plasma, but in this case no bleeding tendency is shown. Quantitative coagulation factor assays should be also performed which reveal a reduced level of FVIII:C in AHA. Milder reduction of factor IX, XI, and XII plasma levels may also be shown; increasing dilutions of patient plasma with normal plasma lead to diluting the autoantibody and to normalizing activities of factors other than FVIII, which remains inhibited even by the diluted specific autoantibody. These tests also enable the detection of more rare cases due to antibodies against coagulation factors other than FVIII [6]. The diagnosis is then confirmed by a Bethesda (or its Nijmegen modification) positive assay. It is noteworthy to remark that the mixing test may be easily carried out in all laboratories and allows the identification of patients who require further investigations at a specialized centre.

When AHA is diagnosed, the possible coexistence of an underlying disease responsible for this immunologic complication should be suspected and intensively searched for. In particular, in elderly patients AHA is often secondary to haematologic or solid malignancy or drugs (e.g., antibiotics, phenytoin, methyldopa, interferon, fludarabine, and clopidogrel) [6, 13]. However, about half of the cases are apparently idiopathic although in some patients an underlying disorder may be diagnosed long after the onset of the bleeding abnormality [6, 17], as reported in patient no. 2.

Our case reports confirm the complexity of the management of AHA in elderly patients, in whom the clinical picture is complicated by comorbidities and concomitant drug intake. The latter may facilitate bleeding complications, as occurred in patients no. 1 and 2, or delay the diagnosis, when antiplatelet or anticoagulant drugs are administered. The coexistence in AHA patients, in particular in the elderly, of overt cardiovascular/thromboembolic diseases (see patient no. 1) and/or risk factors such as diabetes mellitus, neoplasm, arterial hypertension, high body mass index, requiring antithrombotic treatment, represents a challenging issue in this setting. The occurrence of severe bleeding leads to the withdrawal of such treatments and in many cases to the administration of bypassing agents (APCCs, rFVIIa), with possible increase of thromboembolic risk. This risk is further enhanced by the reduced mobility due to hospitalization or muscle bleeding or by activation of coagulation induced during sepsis. Thus an accurate balance of bleeding and thrombotic risk should be carefully taken into account when these patients are treated for the bleeding complications, in terms of doses and duration of bypassing agent administration. Presently, however, evidence of rFVIIa and aPCC thrombogenicity is limited, therefore according to recent recommendations, bypassing agents should not be considered contraindicated in the presence of severe or life-threatening bleed and also in patients at thromboembolic risk [1–6]. In elderly patients, however, especially in those presenting posttreatment high FVIII:C levels, an adequate prolonged prophylaxis with antithrombotic agents, following inhibitor eradication, should be considered (see Case 1). 

An early and appropriate therapeutic approach is crucial for a favourable outcome, which also depends on the treatment and the prognosis of any possible concomitant disease or triggering condition [6, 8, 13]. Bleeding complications are fatal in 10%–20% of the cases, but the overall mortality in AHA patients is also higher, in particular, in elderly patients and over the first weeks after the onset of symptoms, because of the underlying associated diseases, diagnostic delays, inadequate treatment of acute bleeds, bleeding complications during invasive procedures for controlling hemorrhages, or adverse events of treatment (infections, sepsis on immunosuppressive therapy, see Case 3) [3–6].

Therefore, patients with AHA should be managed by a hemophilia center with laboratory and clinical experience in this setting, in particular because of the complexity of treatment. Immunosuppressive regimens in the elderly should aim to eradicate the inhibitor as rapidly as possible, reducing the time of exposure to the side effects of immunosuppressive therapy [5]. Prednisone (1 mg/Kg/day for 4–6 weeks) remains the first-line treatment, alone or in combination with cyclophosphamide (1.5–2 mg/Kg/day). The latter should be administered for a maximum of six weeks, and the risk/benefit ratio of immunosuppressive therapy should be taken into account for each elderly patient individually [5]. Other approaches should be considered for refractory patients. In particular, rituximab may be suggested when first-line immunosuppressive treatment fails or is contraindicated [5, 10–14]. 

Despite the lack of definite conclusions on the optimal hemostatic and inhibitor eradicating approaches, a series of effective options is presently available, giving the opportunity of tailoring the treatment of this rare but severe disease, even in the “fragile” elderly patients.

## Figures and Tables

**Table 1:** Diagnosis of AHA in the elderly (see [5] by Huth-Kuhne et al.).

The diagnosis of AHA should be considered in the presence of both
(1) acute/recent bleeding in elderly patients with no previous
history of bleeding,
(2) unexplained isolated aPTT

**Table 2:** AHA: differential diagnosis in the elderly.

(1) Mild-moderate hereditary haemophilia diagnosed after
the age of 60 years [16]
(2) Lupus anticoagulant
(3) Bleeding complications of anticoagulant treatments
(4) Trauma
(5) Abuse of NSAIDs
(6) Other acquired bleeding disorders (acquired von
Willebrand disease, acquired platelet dysfunctions,
uremia, and liver cirrhosis)

**Table 3:** Diagnostic tests in AHA in the elderly (see Huth-Kuhne et al., modified [5]).

(1) *Mixing test*: prolongation of the aPTT in a mixture of
patient and normal plasma after 1-2 h incubation
compared to an immediate mix is typical of FVIII
autoantibodies.
(2) *Clotting factor measurement*: patients should have FVIII,
IX, X, XI, XII levels measured; an isolated low FVIII level
is suggestive of AHA.
(3) *Quantification of the inhibitor titer*: Bethesda assay, the test
should be repeated to confirm the presence of the inhibitor.
